# PHARAO-Studie: Arzneimittelversorgung entzündlich rheumatischer Erkrankungen: Leitliniengerechte Versorgung mit Biologika folgt Verfügbarkeit von Biosimilars

**DOI:** 10.1007/s00393-022-01259-5

**Published:** 2022-08-25

**Authors:** Franziska Hörbrand, Florian Schuch, Hans-Holger Bleß, David Messinger, Bork Bretthauer, Peter Killian

**Affiliations:** 1 Kassenärztliche Vereinigung Bayerns (KVB), München, Deutschland; 2 Rheumatologische Schwerpunktpraxis Erlangen, Erlangen, Deutschland; 3 fbeta GmbH, Akazienstr. 31, 10823 Berlin, Deutschland; 4 Pro Generika e. V., Berlin, Deutschland

**Keywords:** GKV-Routinedaten, Versorgungsforschung, Biologika, Biosimilars, Rheuma, Claims data, Health care research, Biologicals, Biosimilars, Rheumatism

## Abstract

**Hintergrund:**

Mit Einführung der Tumornekrosefaktor(TNF)-α-Blocker hat die Behandlung entzündlich rheumatischer Erkrankungen (ERE) einen grundlegenden Wandel erfahren. Etliche der ursprünglich hochpreisigen Biologika verloren im Verlauf der Studie ihren Patentschutz und standen seitdem als kostengünstigere Biosimilars zur Verfügung, sodass ein bedeutsames Verordnungshemmnis entfallen ist.

**Fragestellung:**

In der vorliegenden Studie wurde untersucht, ob die Verfügbarkeit von Biosimilars mit einer Verbesserung der Versorgung von ERE einhergeht. Zugleich wurde die subjektive Akzeptanz von Biosimilars bei Ärzten und Patienten untersucht und mit standardisierten Scores abgeglichen.

**Material und Methoden:**

Als Datengrundlage dienten pseudonymisierte Abrechnungsdaten der Kassenärztlichen Vereinigung Bayerns von 2014 bis 2019 sowie eine Paper-Pencil-Befragung von Patienten und Rheumatologen.

**Ergebnisse:**

Im Beobachtungszeitraum stieg der Anteil an diagnostizierten Patienten, die eine Arzneimitteltherapie erhielten, von 38,5 % auf 43,2 % an. Deren Versorgung veränderte sich auch in Bezug auf die verordneten Wirkstoffe. Die konventionelle medikamentöse Therapie war insgesamt rückläufig. Insbesondere die Verordnung von Glukokortikoiden sank von 39,3 % in 2014 auf 34,3 % in 2019. Zugleich stieg der Anteil zielgerichteter Behandlungen von 12,3 % auf 20,4 %. Die mediane Dauer der Basistherapie vor erstmaligem bDMARD-Einsatz verkürzte sich von 3,15 Jahren in 2014 auf 2,17 Jahre in 2019.

**Diskussion:**

Über den Beobachtungszeitraum, in den auch der Markteintritt von 3 Biosimilars fällt, verbesserte sich die Versorgung von Patienten mit ERE quantitativ wie qualitativ. Der Versorgungsanteil von Biosimilars nahm parallel zu der aufgezeigten Entwicklung zu. Bei insgesamt hoher Akzeptanz von Biosimilars verweist die Einschätzung des Krankheitsverlaufes von Ärzten und Patienten auf einen leichten, subjektiv wahrgenommenen Vorteil der Therapie mit Originalen im Vergleich zur Biosimilar-Therapie, der sich bei Anwendung standardisierter Scores jedoch nicht bestätigt. Eine mögliche Erklärung hierfür könnte ein Nocebo-Effekt sein, der durch geeignete Kommunikationsstrategien minimiert werden könnte.

## Abstract (en)

**Background:**

With the introduction of tumor necrosis factor (TNF) alpha inhibitors, the treatment of inflammatory rheumatic diseases (IRD) has undergone a fundamental change. Several of the originally high-priced biologics are now accessible as lower cost biosimilars, removing a significant impediment to prescription.

**Objective:**

The present study investigated whether the availability of biosimilars is associated with an improvement in the care of IRD. Moreover, the subjective acceptance of biosimilars by physicians and patients was investigated and compared with objectifiable parameters.

**Material and methods:**

Pseudonymized claims data of the Bavarian Association of Statutory Health Insurance Physicians from 2014 to 2019 as well as a paper and pencil survey of patients and rheumatologists formed the data basis of the study.

**Results:**

During the observation period, the proportion of diagnosed patients receiving drug therapy increased from 38.5% to 43.2%. Also, the care changed in terms of the prescribed agents. Conventional drug therapy declined overall and, in particular, glucocorticoid prescriptions decreased from 39.3% in 2014 to 34.3% in 2019. At the same time, the proportion of targeted treatments increased from 12.3% to 20.4%. The median duration of basic treatment before first-time bDMARD use dropped from 3.15 years in 2014 to 2.17 years in 2019.

**Conclusion:**

Over the observation period, in which three biosimilars entered the market, the care of patients with IRD improved both quantitatively and qualitatively. The market share of biosimilars increased in parallel with this development. With an overall high acceptance of biosimilars, the assessment of the disease course by physicians and patients indicates a slight subjectively perceived advantage of therapy with originals compared to biosimilars, which, however, is not confirmed when standardized scores are applied. A possible explanation for this might be a nocebo effect, which could be minimized by suitable communication strategies.

Die Behandlung von Menschen mit entzündlich rheumatischen Erkrankungen (ERE) hat in den letzten beiden Jahrzehnten einen grundlegenden Wandel erlebt. Nachdem die Therapiemöglichkeiten lange Zeit mit dem Einsatz von Glukokortikoiden und konventionellen, synthetischen csDMARDs („conventional synthetic disease modifying antirheumatic drugs“) limitiert waren, wurden die Behandlungsoptionen insbesondere schwerer Krankheitsverläufe im Jahr 1999 mit der Zulassung des ersten Biologikums – dem Tumornekrosefaktor(TNF)-α-Blocker Infliximab, und in Folge weiterer Biologika („biologic DMARDs“ [bDMARDs]) – deutlich verbessert. Das Therapieziel ist heute die Remission, also die Abwesenheit von Erkrankungsaktivität und damit das Verhindern von Langzeitfolgen, wie z. B. Gelenkersatzoperationen, aber auch kardiovaskulärer Ereignisse [1]. Etliche der ursprünglich hochpreisigen Biologika stehen seit Ablauf des Patentschutzes als kostengünstigere Biosimilars zur Verfügung [2]. Zudem gilt für Infliximab seit April 2018 ein Festbetrag der Stufe 1 für wirkstoffgleiche Arzneimittel [3]. Für die übrigen TNF-α-Blocker gilt seit April 2021 ein Festbetrag der Stufe 2, der pharmakologisch und therapeutisch vergleichbare Wirkstoffe betrifft [4].

## Hintergrund

In der leitliniengerechten Versorgung von ERE sind Biologika inzwischen fest verankert. csDMARDS wie Methotrexat (MTX) werden nach wie vor zur Erstlinientherapie eingesetzt. Nach dem Treat-to-target-Konzept sind bei inadäquatem Ansprechen eine Therapieintensivierung und bei hoher Krankheitsaktivität bzw. Vorliegen ungünstiger Prognosefaktoren bDMARDs indiziert. Dies ist dann der Fall, wenn 3 Monate nach Therapiebeginn keine Verbesserung bzw. nach 6 Monaten keine Remission erreicht wird [5, 6].

Der Einsatz von Biologika bei rheumatischen Erkrankungen erfolgte zunächst eher zurückhaltend. Neben der mangelnden Erfahrung und der zu Beginn fehlenden Evidenz zur Langzeitsicherheit der TNF-Inhibition wurden auch die hohen Kosten der Arzneimittel und die damit einhergehende Regressangst seitens der Ärzteschaft immer wieder als Ursache genannt [7, 8].

Mit Ablauf des Patentschutzes für die Wirkstoffe Infliximab, Etanercept und Adalimumab in den Jahren 2015 bis 2018 wurden für die umsatzstärksten TNF-α-Blocker günstigere Biosimilars in gleicher Wirksamkeit, Qualität und Sicherheit zugelassen. Damit ist ein bedeutsam erscheinender Grund für den zurückhaltenden Einsatz von Biologika entfallen. Bei einem deutschlandweiten Jahresumsatz der 3 betreffenden bDMARDs von knapp 1,6 Mrd. € im Jahr 2020 wird in der Kassenärztlichen Vereinigung Bayerns (KVB) bereits ein Großteil der Tagesdosen („defined daily doses“ [DDD]) der betreffenden bDMARDs als Biosimilars verordnet (Stand im 1. Halbjahr 2021: Adalimumab 74,7 %, Etanercept 85,6 %, Infliximab 81,8 %) [9, 10].

## Ziel der Studie

In der vorliegenden Studie wurde untersucht, inwiefern die Verfügbarkeit von Biosimilars das Verordnungsverhalten in der ambulanten vertragsärztlichen Versorgung Bayerns verändert hat und ob dies mit einer Verbesserung der Versorgung von Menschen mit ERE (rheumatoide Arthritis, axiale Spondyloarthritis und Psoriasisarthritis) einherging.

Parallel dazu wurden das ärztliche Therapieverhalten und die Krankheitsbelastung bei ERE hinsichtlich des Einsatzes von biologischen Originalen und Biosimilars beleuchtet.

## Material und Methoden

§ 287 SGB V ermöglicht den Kassenärztlichen Vereinigungen (KV), unter definierten Rahmenbedingungen (KV-Kodex) Projekte zur Versorgungsforschung in Zusammenarbeit mit der Pharmaindustrie durchzuführen (PHARAO – Pharma Analyse Recherchen Arznei Objektivität) [11].

### Routinedatenanalyse

Die KVB vertritt rund 28.000 niedergelassene Ärzte und Psychotherapeuten. Der retrospektiven Querschnittanalyse liegen Abrechnungsdaten der gesetzlich Versicherten im Bereich der KVB von 2014 bis 2019 zugrunde. Die Analyse deckt den Zeitraum von 1 Jahr vor Markteintritt des ersten Biosimilars zu Infliximab bis hin zu 1 Jahr nach Einführung des Biosimilars zu Adalimumab ab. Der Datenkörper umfasst alle in der Gesetzlichen Krankenversicherung (GKV) Versicherten, die im Untersuchungszeitraum in Bayern mindestens einen Arztkontakt im Rahmen des vertragsärztlichen Leistungsgeschehens hatten. Hierbei handelt es sich um die vertragsärztlichen Abrechnungs- und Arzneimitteldaten gemäß § 295 und § 300 SGB V. Diese Daten sind pseudonymisiert und umfassen Alter und Geschlecht der Patienten, Angaben zur Verschreibung (Fachrichtung des verordnenden Arztes, Wirkstoff, Verordnungsmonat, Dosierung, Anzahl der Packungen und Darreichungsformen) sowie Angaben zu den Diagnosen nach der Internationalen Klassifikation der Krankheiten, 10. Auflage (ICD-10).

Die Falldefinition erfolgte anhand des ICD-10-GM-Katalogs (German Modification) mit dem sog. M2Q-Kriterium (mindestens 2 Quartale). Hierbei handelt es sich um das Aufgreifkriterium aus dem morbiditätsorientierten Risikostrukturausgleich (Morbi-RSA) zur Identifikation von Patienten mit definierten Krankheitsbildern [12]. Das Kriterium beinhaltet, dass mindestens in einem Quartal des Jahres eine gesicherte Diagnose und eine zweite in einem der 3 auf das Indexquartal folgenden Quartale gestellt wurde. Eingeschlossen wurden die Diagnosen für

- rheumatoide Arthritis (chronische Polyarthritis [RA]) M05.-, M06.-, M08.-, M13.-,
- axiale Spondyloarthritis (axSpA) M45.-,
- Psoriasisarthritis (PSA) M07.-, M09.-.

Die Einteilung der Arzneimitteltherapie erfolgte anhand folgender Wirkstoffgruppen:

- Glukokortikoide,
- csDMARDs (Methotrexat, Leflunomid, Sulfasalazin, Hydroxychloroquin, Azathioprin, Mycophenolsäure, Ciclosporin, Cyclophosphamid),
- bDMARDs mit dem Wirkprinzip TNF-α-Blocker (Adalimumab, Certolizumab pegol, Etanercept, Golimumab, Infliximab),
- bDMARDs mit anderen Wirkprinzipien (Anti-CD20/Rituximab, Anti-CTLA-4/Abatacept, Interleukin-Inhibitor/Sarilumab, Tocilizumab),
- JAK-Inhibitoren („targeted synthetic DMARDs“ [tsDMARDs]) wie Baricitinib und Tofacitinib.

### Paper-Pencil-Befragung

Weiterhin wurde eine Paper-Pencil-Befragung von Patienten und Rheumatologen zur Therapie bei ERE durchgeführt.

Gegenstand der Untersuchung waren der Gesundheitszustand und die gesundheitsbezogene Lebensqualität bei einer Therapie mit biologischem Original im Vergleich zu Biosimilars anhand von:

- Einschätzungen des Krankheitszustands seitens der Patienten und der Ärzte seit Beginn der biologischen Therapie und seit dem letzten Praxisbesuch bzw. Patientenkontakt,
- den zuletzt erhobenen standardisierten Scores des krankheitsbezogenen Gesundheitszustandes,
- aktueller und, sofern zutreffend, vergangener biologischer Therapie (Original und Biosimilar),
- den Therapiezeiträumen vor und nach Beginn der Behandlung mit dem aktuellen Biologikum/Biosimilar sowie
- soziodemografischen Patientendaten.

Die Stichprobengröße betrug 977 Patientendokumentationen von 25 bayerischen vertragsärztlich tätigen Rheumatologen, die folgende Kriterien erfüllt haben:

- Patient hat Diagnose rheumatoide Arthritis, axiale Spondyloarthritis oder Psoriasisarthritis,
- Arzt sieht Patient mindestens zum zweiten Mal und
- Patient erhält bereits oder ab sofort eine Arzneimitteltherapie mit einem bDMARD (Original oder Biosimilar).

Die Fragebogenentwicklung erfolgte in Zusammenarbeit der KVB und Pro Generika in enger Abstimmung mit einem rheumatologischen Experten. Die Auswertung des Fragebogens erfolgte durch die DocCheck Research, Köln. Die Patientendokumentationen wurden durch die Ärzte gemeinsam mit den Patienten im Zeitraum 24.10.19 bis 31.01.2020 während der Sprechstunde ausgefüllt. Je Patient wurde eine Erhebung durchgeführt.

## Ergebnisse

### Steigende Verordnungshäufigkeit zielgerichteter Therapien

**Figure 1:**
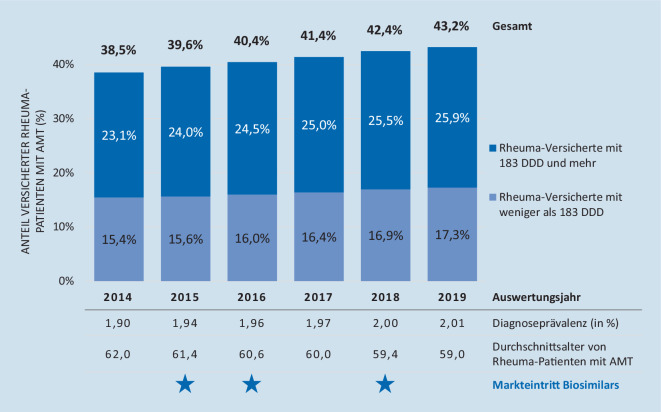

Im Zeitraum 2014 bis 2019 ist ein Anstieg der Diagnoseprävalenz von rheumatischen Erkrankungen von 1,9 % auf 2,01 % in Bayern zu beobachten (s. Abb. 1, vgl. auch Tab. 1). Auch die Arzneimittelversorgung dieser Patienten nahm von 2014 bis 2019 in Bayern zu. Über diesen Zeitraum stieg bayernweit der Anteil an diagnostizierten Patienten, die eine Arzneimitteltherapie (AMT) erhielten, von 38,5 % auf 43,2 % (s. Abb. 1) bei gleichzeitig sinkendem durchschnittlichem Alter von 62 Jahren in 2014 auf 59 Jahre in 2019. Auch der Anteil von Patienten unter Dauertherapie, die mehr als 183 DDD jährlich erhielten, nahm zu (2014: 23,1 %; 2019: 25,9 %). Der morbiditätsbezogene Risikostrukturausgleich (Morbi-RSA) definiert 183 DDD als Mindestmenge für eine Dauermedikation bei Versicherten mit chronischer Krankheit [12].^1^

**Table 1:** 

		Männlich (%)	Weiblich (%)	Anteil an Gesamtkohorte (%)
*2014*	*Gesamt (n* *=* *77.818)*	*32*	*68*	*100*
	Rheumatoide Arthritis	29	71	84,2
	Axiale Spondyloarthritis	64	36	4,2
	Psoriasisarthritis	51	49	3,5
	Mischdiagnosen	44	56	8,1
*2019*	*Gesamt (n* *=* *97.131)*	*33*	*67*	*100*
	Rheumatoide Arthritis	30	70	81,3
	Axiale Spondyloarthritis	62	38	4,8
	Psoriasisarthritis	51	49	4,3
	Mischdiagnosen	42	58	9,5

Mischdiagnosen: Es lagen mindestens 2 der 3 ausgewiesenen Diagnosen gleichzeitig vor

Insgesamt ist eine vermehrte Arzneimitteltherapie für gesetzlich versicherte Patienten mit rheumatischen Erkrankungen zu beobachten. Dieser Trend fällt zusammen mit dem Markteintritt von 3 Biosimilars: dem Biosimilar zu Infliximab in 2015, dem Biosimilar zu Etanercept in 2016 und dem Biosimilar zu Adalimumab in 2018.

**Figure 2:**
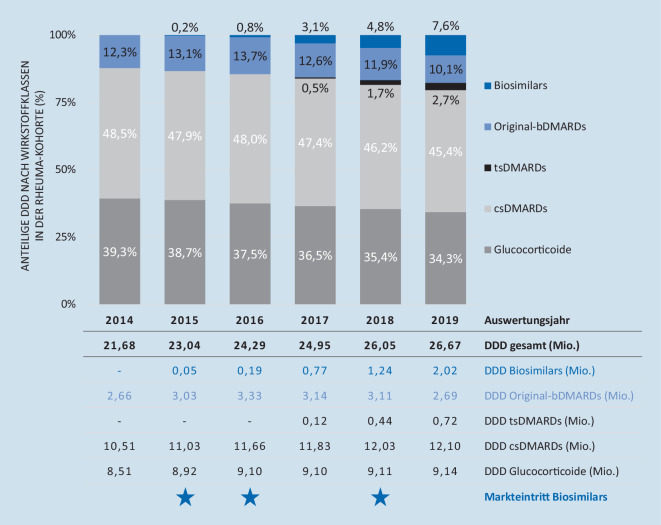

Die Versorgung der Patienten veränderte sich zudem in Bezug auf die verordneten Wirkstoffe. Die konventionelle medikamentöse Therapie rheumatischer Erkrankungen macht weiterhin einen Großteil der Medikation aus. Dieser ist im Untersuchungszeitraum jedoch kontinuierlich gesunken (2014 = 87,7 % vs. 2019 = 79,7 %; s. Abb. 2). Die rückläufige Entwicklung ist sowohl hinsichtlich der anteiligen Versorgung mit csDMARDS als auch im Bereich der kortisonbasierten Therapie zu beobachten (Anteil csDMARDS an DDD-Gesamtvolumen in 2014 = 48,5 % vs. 2019 = 45,4 %; Anteil Glukokortikoide in 2014 = 39,3 % vs. 2019 = 34,3 %).

Im Gegenzug nahm der Anteil zielgerichteter Behandlungen über den Auswertungszeitraum kontinuierlich zu: von 12,3 % in 2014 auf 20,4 % in 2019 für bDMARDs (Originale = 10,1 % und Biosimilars = 7,6 %) und tsDMARDS (2,7 %) zusammen. Jedoch spielten Letztere unter den zielgerichteten Therapien quantitativ noch eine deutlich untergeordnete Rolle. Diese Entwicklung zugunsten leitliniengerechter zielgerichteter Versorgung ging einher mit einem stetig zunehmenden Stellenwert von Biosimilars, die nicht nur konventionelle Therapieoptionen verdrängten, sondern auch Original-bDMARDs – sowohl in absoluten Zahlen als auch anteilig (Anteil Originale 2014 = 12,3 %; 2019 = 10,1 %).

**Figure 3:**
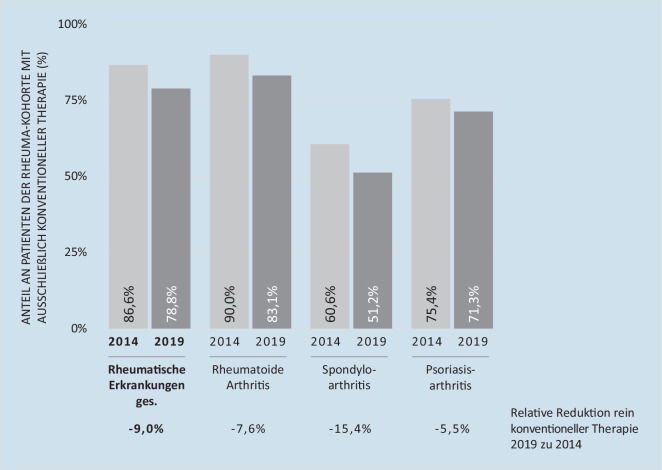

Dieser Trend auf Populationsebene lässt sich auch in Bezug auf patientenindividuelle Medikationsmuster beobachten. So nahm der Anteil an Patienten, die ausschließlich eine konventionelle rheumatische Therapie (Glukokortikoide und/oder csDMARDs) erhielten, von 2014 zu 2019 innerhalb der untersuchten Kohorte über alle rheumatischen Erkrankungen um 9,0 % ab (s. Abb. 3). Der stärkste Rückgang ist bei der axialen Spondyloarthritis zu verzeichnen (−15,4 %).

**Figure 4:**
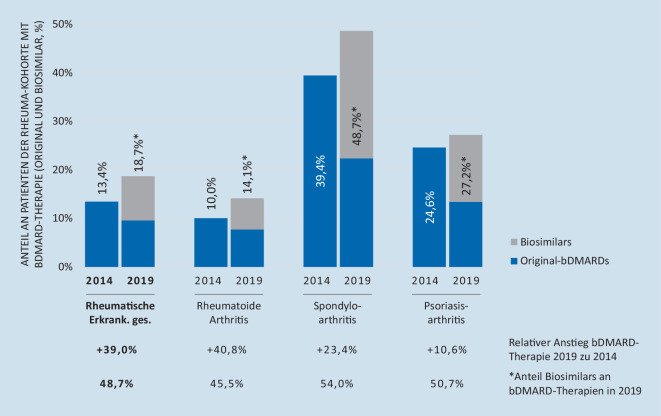

Im Vergleich dazu stieg der Anteil an Patienten mit einer biologischen Therapie (Originale und/oder Biosimilars) über denselben Zeitraum deutlich (s. Abb. 4). Der stärkste Anstieg ist mit 40,8 % bei der Versorgung von Patienten mit rheumatoider Arthritis festzustellen. Während in 2014 noch keine Biosimilars auf dem Markt verfügbar waren, erhielt in 2019 über alle rheumatologischen Erkrankungen hinweg bereits fast die Hälfte (48,7 %) der mit biologischen Therapien versorgten Patienten eine Biosimilar-Therapie.

Zielgerichtete Therapien und somit auch bDMARDs werden hauptsächlich durch Rheumatologen verordnet: Im Zeitraum von 2014 bis 2019 waren Rheumatologen für 74,7 % und andere Facharztgruppen (z. B. Orthopäden) für weitere 13,8 % der verordneten zielgerichteten Therapien verantwortlich (bDMARDs: 74,1 % und 14,3 %).^2^ Rheumatologen sind damit die Arztgruppe, deren Therapieverhalten maßgeblich Rückschlüsse über den Einfluss der Einführung von Biosimilars auf die ambulante vertragsärztliche Versorgung zulässt.

Wie anhand der KVB-Abrechnungsdaten gezeigt, nahm der Anteil an Biosimilars gegenüber Original-bDMARDs in der Versorgung von 2014 bis 2019 zu. Aus den Patientendokumentationen der Paper-Pencil-Befragung mit niedergelassenen Rheumatologen geht hervor, dass zum Zeitpunkt der Erhebung mehr Patienten eine Therapie mit Originalen als mit Biosimilars erhielten (56 % zu 44 %, s. Tab. 2). Wurde jedoch die rheumatische bDMARD-Therapie umgestellt, fand ein Wechsel von Original auf Biosimilar mit 39 % deutlich häufiger statt als umgekehrt (7 %, s. Tab. 3).

**Table 2:** 

	Männlich (%)	Weiblich (%)	Gesamt (%)
*bDMARD-Original*-behandelte Patienten (*n* = 544)	38	62	**56**
***Biosimilar***-behandelte Patienten (*n* = 422)	45	55	**44**
**Gesamt** bDMARD-behandelte Patienten (*n* = 966)	**41**	**59**	**100**

*bDMARD* Biologic disease-modifying anti-rheumatic drugs

**Table 3:** 

	Umstellung *von Original*		Umstellung **von Biosimilar**	
	*Auf Original* (%)	***Auf Biosimilar ***(%)	*Auf Original *(%)	***Auf Biosimilar ***(%)
RA (*n* = 316)	45	37	8	10
PSA (*n* = 120)	51	36	7	7
axSpA (*n* = 124)	42	46	6	6
**ERE gesamt (*n*** **=** **560)**	**45**	**39**	**7**	**9**

*RA* Rheumatoide Arthritis, *PSA* Psoriasisarthritits, *axSpA* axiale Spondyloarthritis

Die Paper-Pencil-Befragung zeigte einen zunehmend früheren Einsatz von bDMARDs. So betrug die mediane Dauer der Basistherapie (Glukokortikoide und csDMARDs) von Patienten, die bis einschließlich 2013 erstmals auf eine bDMARD-Therapie eingestellt wurden, noch 3,71 Jahre (s. Tab. 4). Im Zeitraum ab 2014, in den die Markteinführungen von 3 Biosimilars fielen, sank die Dauer der Basistherapie vor Biologikaeinsatz mit Ausnahme von 2018 jedes Jahr weiter ab. Für Patienten, deren Ersteinstellung auf bDMARD 2019 erfolgte, verkürzte sich die mediane Dauer der Basistherapie auf 2,17 Jahre.

**Table 4:** 

Jahr der Ersteinstellung auf ein bDMARD	Bis 2013	2014	2015	2016	2017	2018	2019
Mediane Dauer der Basistherapie bis zur Ersteinstellung (Jahre)	3,71	3,15	2,57	2,46	2,25	2,66	2,17
Anzahl dokumentierter Fälle (*n*)	339	76	88	99	115	101	133

### Wahrnehmung der Wirksamkeit von Biosimilars und Originalen vergleichbar

**Figure 5:**
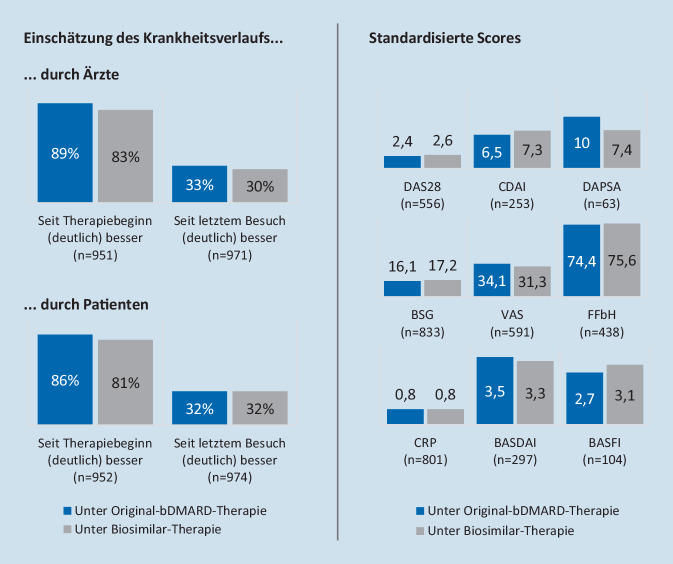

Rheumatologen schätzten den Krankheitsverlauf bei Patienten, die eine Original-bDMARD-Therapie erhielten, im Einzelfall etwas besser ein als bei Patienten unter Biosimilars. So gaben Behandler in der Paper-Pencil-Befragung bei 89 % der mit Originalen behandelten Patienten an, der Gesundheitszustand habe sich seit Beginn der aktuellen Therapie (deutlich) verbessert (33 % seit dem letzten Besuch; s. Abb. 5, linke Seite). Diese Einschätzung traf für 83 % (30 %) der mit Biosimilars behandelten Patienten zu. Ähnliche Unterschiede lieferte auch die Selbsteinschätzung von Patienten (86 % vs. 81 % seit Therapiebeginn; 32 % vs. 32 % seit dem letzten Besuch Original vs. Biosimilar).

Zusätzlich zur Einschätzung des Krankheitszustands wurden im Rahmen der Umfrage auch die letzterhobenen standardisierten Scores der Patienten dokumentiert. Während einige Scores zugunsten der Therapie mit Originalen ausschlugen (z. B. DAS28, CDAI, BASFI), erzielten bei anderen Biosimilars die besseren Werte (z. B. DAPSA, VAS, BASDAI). Insgesamt scheinen die Ergebnisse zwischen den Scores jedoch vergleichbar (s. Abb. 5, rechte Seite).

## Diskussion

Die vorliegende Querschnittanalyse zeigt für den Zeitraum von 2014 bis 2019 eine deutliche Veränderung der medikamentösen Versorgung von Patienten mit ERE. Mit dem Markteintritt von Biosimilars für die umsatzstärksten bDMARDs Adalimumab, Etanercept und Infliximab in den Jahren 2015 bis 2018 standen bei bestehender Indikation für TNF-α-Blocker kostengünstigere Therapieoptionen zur Verfügung.^3^ Damit ging ein häufigerer und früherer Einsatz von bDMARDs im ärztlichen Verordnungsverhalten in Bayern einher, wie er auch bereits für das Rheuma-Register RABBIT beschrieben worden ist [13].

So stieg die Anzahl der Rheumapatienten mit Pharmakotherapie im Beobachtungszeitraum um 24,7 % von 38 % im Jahr 2014 auf 43 % im Jahr 2019. Dabei waren die insgesamt je Patient verordneten Mengen nahezu unverändert, das durchschnittliche Alter der Patienten mit Arzneimitteltherapie sank jedoch zwischen 2014 und 2019 um durchschnittlich 3 Jahre. Gleichzeitig ist ein Anstieg der Patienten mit ERE insgesamt zu beobachten, was sich mit vorangegangenen Studien deckt [14]. Der Anteil der Glukokortikoide an den Gesamt-DDD aller eingesetzten Wirkstoffklassen ist von 2014 bis 2019 um 5 % gesunken. Es ist also von einem reduzierten Einsatz auszugehen, wie er in den Leitlinien empfohlen wird [2]. Dennoch machen Glukokortikoide immer noch mehr als ein Drittel der verordneten DDD aus (34,3 % in 2019).

Über den Zeitraum von 2014 bis 2019, in den auch der Markteintritt von 3 Biosimilars fällt, scheint sich die Versorgung von Patienten mit rheumatischen Erkrankungen also quantitativ wie qualitativ verbessert zu haben: Mehr Patienten erhielten eine Arzneimitteltherapie, gleichzeitig nahm der Anteil zielgerichteter Therapien zu, und immer weniger Patienten wurden ausschließlich konventionell behandelt. Zudem sank die mediane Dauer der Basistherapie bis zur erstmaligen Umstellung auf eine bDMARD-Therapie von 2014 bis 2019 von 3,15 Jahren auf 2,17 Jahre. Diese Beobachtung ist vorrangig für Patienten mit rheumatoider Arthritis oder Psoriasisarthritis von Bedeutung. Für Patienten mit axialer Spondyloarthritis ist in der Regel eine Biologikatherapie bereits dann indiziert, wenn unter nichtsteroidalen Antirheumatika (NSAR) keine ausreichende Reduktion der entzündlichen Krankheitsaktivität erreicht werden kann [15].

Diese für Betroffene positive Entwicklung zugunsten leitliniengerechter zielgerichteter Versorgung ging einher mit einem stetig zunehmenden Stellenwert von Biosimilars, die nicht nur konventionelle Therapieoptionen verdrängten, sondern auch Original-bDMARDs – sowohl in absoluten Zahlen als auch anteilig (Anteil Originale 2014 = 12,3 %; 2019 = 10,1 %). Dies zeigen auch die Auswertungen des RABBIT-Registers [13].

Der verbesserte Zugang zur Versorgung mit bDMARDs sowie die erhöhte Leitlinienkonformität der Versorgung von entzündlich rheumatischen Erkrankungen stehen im zeitlichen Zusammenhang mit der Möglichkeit einer wirtschaftlichen Verordnung durch Biosimilars. Diese beobachtete Korrelation entspricht der bereits 2017 von der Deutschen Gesellschaft für Rheumatologie aufgestellten Forderung: „*Die Verfügbarkeit von Biosimilars muss die Behandlungskosten von individuellen Patienten senken und den Zugang zu einer optimalen Therapie für alle Patienten mit rheumatischen Erkrankungen ermöglichen“* [16].

Besonderes Augenmerk verdient der Befund, dass Patienten und Ärzte den Krankheitsverlauf beim Einsatz von Originalprodukten etwas positiver einschätzten als beim Einsatz von Biosimilars. Die Ergebnisse der standardisierten Scores hingegen waren vergleichbar.

Dieser Unterschied kann zum einen daher rühren, dass eine subjektive Verbesserung im Krankheitsverlauf nicht zwangsläufig mit einem besseren Score korreliert. Im Alltag können Patienten von Originalpräparaten auf das entsprechende Biosimilar umgestellt werden, was nicht mit dem Ziel einer besseren Wirksamkeit verknüpft ist.

Zum anderen bestätigen auch frühere Untersuchungen die Diskrepanz zwischen der Selbsteinschätzung des Krankheitsverlaufs und der Messung anhand standardisierter Krankheitsparameter [17]. Diese abweichende Wahrnehmung wird mitunter durch den Nocebo-Effekt erklärt [18, 19], der allerdings durch geeignete Kommunikationsstrategien minimiert werden kann [20, 21]. In diesem Zusammenhang können die gefundenen Ergebnisse auf die Notwendigkeit verweisen, Nocebo-induzierende Kommunikation bei der Verordnung von und insbesondere bei Umstellung auf Biosimilars zu vermeiden, wie es auch die Arzneimittelkommission der deutschen Ärzteschaft (AkdÄ) in ihrem Leitfaden zu Biosimilars empfiehlt [22]. Eine derartige Kommunikation ist allerdings zeitaufwendig, was sich vor dem Hintergrund der bekannten personellen Engpässe in der Ärzteschaft und damit auch bei Rheumatologen hemmend auswirken kann.

Aus den Ergebnissen dieser Querschnittanalyse wird deutlich, dass mittlerweile die Akzeptanz der Rheumatologen für den Einsatz von Biosimilars insgesamt hoch ist. Fördernde Faktoren sind dabei auch in den Bestrebungen der Selbstverwaltung in Bayern zu verorten, den Einsatz von Biosimilars durch Maßnahmen wie Biosimilarquoten zu steigern. Diese führten in den zurückliegenden Jahren zu einer hohen Biosimilarquote im bundesweiten Vergleich [23].

Diese Studie unterliegt gewissen Limitationen. So werden GKV-Routinedaten zu Abrechnungszwecken erhoben und spiegeln daher ausschließlich die gegenüber der GKV abgerechneten Leistungen wider, was eine lediglich indirekte Schlussfolgerung auf die in der Versorgung erbrachten Leistungen ermöglicht. Die Auswahl der Patienten für die Paper-Pencil-Befragung erfolgte durch die behandelnden Rheumatologen, womit ein Selektionsbias einhergehen kann. In den Patientendokumentationen wurden die Gründe für Therapieumstellungen nicht erfasst. Beides schränkt die Aussagekraft der Arzt- und Patienteneinschätzung hinsichtlich der Therapie mit biologischen Originalen oder Biosimilars ein. Schließlich sind die zeitlichen Zusammenhänge zwischen der Einführung von Biosimilars und den beobachteten Effekten als Korrelation beobachtet worden und bedingen sich nicht zwingend kausal.

## Fazit für die Praxis

- Biologika gewinnen aufgrund ihrer zielgerichteten Therapieansätze in der individuellen Patientenversorgung immer mehr an Bedeutung. Bei in der Regel guter Verträglichkeit und Langzeitsicherheitsdaten über mehr als 20 Jahre für TNF-α-Blocker ermöglichen diese Therapien bei vielen Patienten das Therapieziel der Remission der Erkrankung. Sobald diese in der Regel teureren Therapieoptionen den Patentschutz verlieren, erschließen sich wirtschaftliche Reserven.
- Bei funktionierender Verordnungssteuerung durch die Selbstverwaltung und hoher ärztlicher Akzeptanz von Biosimilars sollte sich die weitere Förderung des Einsatzes von Biosimilars auf eine verbesserte Patientenkommunikation konzentrieren. Hier sind incentivierende Steuerungsansätze vorstellbar, um sowohl das kostensenkende als auch das versorgungsverbessernde Potenzial der Biosimilars zu realisieren. Steuerungsmechanismen, wie die gesetzlich vorgesehene Substitution von Biosimilars in der Apotheke, mögen zwar die gewünschten Auswirkungen auf (weitere) Rabatte entfalten und die Erhöhung des Verordnungsanteils rabattierter Biologika befördern. Anstelle der benötigten Verbesserung der Patientenkommunikation könnte eine Verunsicherung der Patienten resultieren, die die erzielten Qualitätsverbesserungen in deren Versorgung infrage stellen würde.

## Footnotes

1 Für eine konsistente Betrachtung der gesamten Rheuma-Kohorte über alle Auswertungen beinhaltet die Auswertung der DDD auch Kinder und Jugendliche mit AMT. Für diese Patientengruppe sind DDD nicht anwendbar. Da der Anteil an Kindern und Jugendlichen in der Kohorte mit 0,47 % (2014) und 0,67 % (2019) gering ist und lediglich im Nachkommabereich die Gesamtverteilung und das Durchschnittsalter beeinflusst, wurde auf eine separate Darstellung verzichtet.

2 Zielgerichtete Therapien = bDMARDs (Originale und Biosimilars) + tsDMARDs; Angaben basierend auf verordneten DDD nach Wirkstoff und Arztgruppe, Quelle: KVB-Datenbank, August 2020.

3 Die ausgewerteten Daten beziehen sich auf den Zeitraum vor der Bildung einer Festbetragsgruppe für TNF-α-Blocker in 2020.

## References

[1] Burmester GR, Pope JE (2017). Novel treatment strategies in rheumatoid arthritis. Lancet.

[2] Dicheva-Radev S, Ludwig DW, Schwabe U (2019). Biologika und Biosimilars. Arzneiverordnungs-Report.

[3] GKV-Spitzenverband (2018). Bekanntmachung von Beschlüssen des Spitzenverbandes Bund der Krankenkassen nach § 35 SGB V.

[4] GKV-Spitzenverband (2021). Bekanntmachung von Beschlüssen des Spitzenverbandes Bund der Krankenkassen nach § 35 SGB V.

[5] Schneider M (2020). Management der frühen rheumatoiden Arthritis. Z Rheumatol.

[6] Smolen JS (2020). EULAR recommendations for the management of rheumatoid arthritis with synthetic and biological disease-modifying antirheumatic drugs: 2019 update. Ann Rheum Dis.

[7] Osterloh F (2019) Experten für Vergütung von Arztgesprächen zur Umstellung auf Biosimilars. Aerzteblatt.de. https://www.aerzteblatt.de/nachrichten/100572/Experten-fuer-Verguetung-von-Arztgespraechen-zur-Umstellung-auf-Biosimilars. Zugegriffen: 31. Jan. 2021

[8] Meißner T (2007) Weil es zu wenige Rheumatologen gibt, wird die Krankheit oft spät erkannt – das schadet nicht nur den Patienten. ÄrzteZeitung. https://www.aerztezeitung.de/Medizin/Weil-es-zu-wenige-Rheumatologen-gibt-wird-die-Krankheit-oft-spaet-erkannt-das-schadet-nicht-nur-de-393340.html. Zugegriffen: 31. Jan. 2021

[9] GKV-Spitzenverband (2021). GKV-Arzneimittel-Schnellinformation für Deutschland nach § 84 Abs. 5 SGB V.

[10] GKV-Spitzenverband (2021). GKV-Arzneimittel-Schnellinformation für die KV Bayern nach § 84 Abs. 5 SGB V.

[11] https://www.kvb.de/service/partner/versorgungsforschung/pharma-kooperation-pharao/. Zugegriffen: 17. Dez. 2021

[12] Bundesversicherungsamt (2013). Festlegungen nach § 31 Abs. 4 RSAV für das Ausgleichsjahr 2014.

[13] Strangfeld A (2021). Biosimilars in den deutschen Biologika-Registern RABBIT und RABBIT-SpA. Kompendium Biosimilars.

[14] Steffen A (2017). Epidemiology of rheumatoid arthritis in Germany—an analysis based on nationwide claims data of outpatient care. Central Research Institute of Ambulatory Health Care in Germany (Zi).

[15] Kiltz U (2019). DGRh-S3-Leitlinie Axiale Spondyloarthritis inklusive Morbus Bechterew und Frühformen.

[16] Braun J (2018). Neufassung der Stellungnahme der DGRh zu Biosimilars – Update 2017. Z Rheumatol.

[17] Kravvariti E (2018). Nocebos in rheumatology: emerging concepts and their implications for clinical practice. Nat Rev Rheumatol.

[18] Fleischmann R (2020). Nonmedical switching from originators to biosimilars: does the nocebo effect explain treatment failures and adverse events in rheumatology and gastroenterology?. Rheumatol Ther.

[19] Bakalos G, Zintzaras E (2019). Drug discontinuation in studies including a switch from an originator to a biosimilar mono-clonal antibody: a systematic literature review. Clin Ther.

[20] Colloca L (2019). The clinical implications of nocebo effects for biosimilar therapy. Front Pharmacol.

[21] Tweehuysen L (2017). FRI0200 Higher acceptance and persistence rates after biosimilar transitioning in patients with a rheumatic disease after employing an enhanced communication strategy. Ann Rheum Dis.

[22] Arzneimittelkommission der deutschen Ärzteschaft (2021). Leitfaden „Biosimilars“. 2. Auflage, Version 1.0.

[23] Zentralinstitut für die kassenärztliche Versorgung in Deutschland Zi-Arzneiverordnungsdaten-Portal. https://www.zi.de/projekte/analysetools/zi-arzneiverordnungs-portal. Zugegriffen: 19. Aug. 2021

